# The Association Between Sleep and Obesity in Adults: An Umbrella Review of Meta‐Analyses

**DOI:** 10.1002/hsr2.73449

**Published:** 2026-10-27

**Authors:** Iana Malasevskaia, Delvy Rebellow, Pakeeza Tarar, Esraa M. Soliman

**Affiliations:** ^1^ Private Clinic of Obstetrics and Gynecology Sana'a Yemen; ^2^ Malankara Orthodox Syrian Church Medical College Kolenchery Kerala India; ^3^ Allama Iqbal Medical College Lahore Pakistan; ^4^ Faculty of Medicine Zagazig University Zagazig Egypt

**Keywords:** adults, meta‑analysis, obesity, short sleep, sleep duration, umbrella review

## Abstract

**Background and Aims:**

Short sleep duration, poor sleep quality, and circadian disruption have been linked to obesity, but the proliferation of systematic reviews with meta‐analyses (SRMAs) has fragmented the evidence. No prior umbrella review has synthesized all sleep characteristics exclusively in adults or formally graded the credibility of associations. We aimed to evaluate the association between various sleep characteristics and obesity‑related outcomes in adults.

**Methods:**

We conducted an umbrella review of SRMAs. PubMed, Embase, Cochrane Database of Systematic Reviews, Scopus, CINAHL, and Epistemonikos were searched from inception to March 1, 2026. We included SRMAs examining adults (≥ 18 years) reporting any sleep exposure and obesity outcomes. Methodological quality was assessed using AMSTAR 2. Overlap of primary studies was quantified with the corrected covered area (CCA). Evidence was graded using Ioannidis criteria.

**Results:**

Sixteen SRMAs encompassing approximately 13.8 million participants were included. Primary study overlap was very low (overall CCA = 0.024). Short sleep duration was associated with a 40% increased odds of obesity (pooled OR 1.40, 95% CI 1.27–1.55; I^2^ = 83.7%). Long sleep showed a modest association (OR 1.05, 95% CI 1.02–1.08; I^2^ = 0%). Both were classified as Class III (Suggestive) evidence. Daytime napping, social jetlag, and insomnia symptoms met only Class IV (Weak) criteria. Objective sleep measurement (polysomnography) yielded substantially larger effects (OR 2.70, 95% CI 1.80–4.05) than self‑report (OR 1.14, 95% CI 0.98–1.33). AMSTAR 2 assessment revealed two high‑quality, four moderate, nine low, and one critically low review.

**Conclusions:**

Short and long sleep durations are suggestively associated with obesity in adults, with short sleep showing a stronger effect. Other sleep characteristics have weak or limited evidence. Clinicians should consider sleep duration a potentially modifiable risk factor for obesity.

**PROSPERO Registration:**

CRD420261326462.

**Abbreviations**
AMSTAR 2: A Measurement Tool to Assess Systematic Reviews 2
BMI: Body mass index
CCA: Corrected covered area
CI: Confidence interval
IND: Insomnia diagnosis
INS: Insomnia symptoms
MSF: Mid‐sleep on free days
MSW: Mid‐sleep on work days
OR: Odds ratio
PI: Prediction interval
PRIOR: Preferred Reporting Items for Overviews of Reviews
PRISMA: Preferred Reporting Items for Systematic Reviews and Meta‐Analyzes
PROSPERO: International Prospective Register of Systematic Reviews
RCT: Randomized controlled trial
REML: Restricted maximum likelihood
ROBIS: Risk of Bias in Systematic Reviews
RR: Relative risk
SD: Standard deviation
SMD: Standardized mean difference
SRMA: Systematic review with meta‐analysis
WC: Waist circumference
WHO: World Health Organization
WHR: Waist‐to‐hip ratio

## 1 Introduction

In 2022, 2.5 billion adults worldwide were overweight, of whom 890 million were living with obesity—representing 43% and 16% of the adult population, respectively [1]. Since 1990, adult obesity has more than doubled [1]. This epidemic drives a substantial burden of non‑communicable diseases, including type 2 diabetes, cardiovascular disease, and several cancers, and accounts for approximately 5 million deaths annually [2, 3]. Concurrently, chronic sleep deprivation has become endemic in modern 24‑h societies: over one‑third of adults regularly sleep less than the recommended 7–9 h per night [4]. Poor sleep quality and circadian disruption affect even larger proportions of the population [5]. Sleep is not merely a passive state; it is an active physiological process that regulates metabolism, appetite, and endocrine function [6].

Over the past two decades, numerous systematic reviews with meta‐analyses (SRMAs) have examined the association between sleep and obesity. These have consistently reported that short sleep duration (< 5–7 h/night) is associated with a 30%–50% increased risk of obesity in adults [7, 8, 9]. Long sleep duration (> 8–9 h) has shown a smaller but statistically significant positive association in some reviews [10, 11], while others report null findings [12]. Beyond duration, SRMAs have investigated sleep quality [13], sleep disorders such as insomnia [14] and obstructive sleep apnea [15], and circadian constructs like social jetlag [16]. Outcomes have ranged from BMI‑defined overweight/obesity to central obesity, body fat percentage, and weight gain. Reported effect sizes vary considerably, partly due to differences in exposure definitions, populations, and study quality [17].

The proliferation of SRMAs has created a new challenge: evidence overload. A clinician seeking guidance might find 10 different meta‐analyses on short sleep and obesity, each with slightly different inclusion criteria, effect estimates, and conclusions. This fragmentation obscures the true strength and consistency of the evidence. Importantly, existing SRMAs have limitations that an umbrella review can resolve. First, no single prior overview has synthesized all sleep characteristics and all obesity outcomes exclusively in adults—previous umbrella reviews either focused narrowly on broader health outcomes [18] or combined children and adults [17]. Second, the methodological quality of existing SRMAs has never been systematically appraised using a validated tool. Third, overlap of primary studies across reviews—a known problem in evidence synthesis—has never been quantified for this literature. Fourth, no previous work has applied a formal evidence grading framework to classify the credibility of each association.

To address these gaps, we conducted a comprehensive umbrella review of SRMAs examining the association between sleep characteristics and obesity in adults. Specific objectives were to: (i) systematically identify all relevant SRMAs reporting adult data; (ii) assess their methodological quality using AMSTAR 2 [19]; (iii) quantify overlap of primary studies using the Corrected Covered Area (CCA); (iv) perform a meta‑analysis of meta‑analyzes where appropriate; (v) grade the strength of evidence using the Ioannidis criteria [20]; and (vi) identify evidence gaps and future research priorities. This umbrella review provides a rigorous, high‑level synthesis that: (a) focuses exclusively on adults, (b) covers the full spectrum of sleep exposures, (c) includes formal overlap assessment, (d) applies a validated quality appraisal tool, and (e) grades evidence using established criteria. The findings will help clinicians identify the most robust sleep‑related obesity risk factors and guide researchers towards areas where evidence is still weak or conflicting.

### 1.1 What Was Known/What This Study Adds

#### 1.1.1 What Was Known

Previous systematic reviews and meta‐analyses have reported that short sleep duration is associated with increased obesity risk. However, these reviews used different sleep definitions, focused on single sleep characteristics, and often combined children with adults. The methodological quality of existing reviews and the overlap of primary studies across them had never been systematically assessed.

#### 1.1.2 What This Study Adds

This first umbrella review focused exclusively on adults provides a comprehensive synthesis of all sleep characteristics (duration, quality, napping, social jetlag, insomnia) with formal evidence grading (Ioannidis criteria). Short sleep duration shows suggestive‑level evidence (OR 1.40) with a clear dose‑response gradient. Objective sleep measurement yields substantially larger effects (OR 2.70) than self‑report, suggesting the true association may be underestimated.

## 2 Methods

### 2.1 Protocol Registration

The protocol for this umbrella review was submitted to the International Prospective Register of Systematic Reviews (PROSPERO) on February 26, 2026, and registered on March 2, 2026 (registration number CRD420261326462). No deviations from the registered protocol occurred. The protocol is publicly available. The review was conducted in accordance with established methodological guidance for overviews of reviews [21, 22]. Reporting followed the PRIOR (Preferred Reporting Items for Overviews of Reviews) statement [23], and the study selection process was documented using a PRISMA 2020 flow diagram [24].

### 2.2 Eligibility Criteria

We included SRMAs examining adults (≥ 18 years). Reviews with mixed populations were eligible if adult data were reported separately or constituted the majority (> 80%) of participants. Eligible exposures encompassed any sleep‑related characteristic, including sleep duration, quality, disorders, and chronotype. Outcomes included measures of obesity or adiposity, such as body mass index (BMI), waist circumference, body fat percentage, and incident overweight or obesity. Only reviews reporting quantitative synthesis with pooled effect estimates and measures of heterogeneity were included. Narrative syntheses, reviews lacking meta‑analysis, non‑English full texts, and studies focused exclusively on pediatric populations were excluded.

### 2.3 Search Strategy

A comprehensive search was conducted across PubMed/MEDLINE, Embase, Cochrane Database of Systematic Reviews, Scopus, CINAHL, and Epistemonikos [25] from inception to March 1, 2026, with no date restrictions [26]. The full search strategy is provided in Supplementary Table S1. Searches were supplemented by screening PROSPERO for ongoing reviews and by manual checking of reference lists.

### 2.4 Study Selection and Data Extraction

All identified records were imported into Rayyan, a web‑based systematic review screening platform [27]. Study selection was performed independently by two reviewers (D.R. and P.T.) in two stages: title/abstract screening followed by full‑text assessment. Disagreements were resolved through discussion with a third reviewer (I.M. or E.S.). Reasons for exclusion at the full‑text stage were recorded.

Data extraction was conducted independently by two reviewers (D.R. and P.T.) using a standardized and piloted form. Extracted data included review characteristics, participant characteristics, definitions of exposures and outcomes, pooled effect estimates with 95% confidence intervals, and measures of heterogeneity. When multiple estimates were reported for the same exposure–outcome pair, the most comprehensive or primary estimate was selected: the primary estimate was defined as the main analysis reported by the authors, and the most comprehensive estimate was defined as the analysis with the largest number of primary studies. When both were available, the primary estimate was prioritized. Dose‑response data were extracted from SRMAs where reported; no additional dose‑response analyses were performed.

### 2.5 Methodological Quality Assessment

The methodological quality of included reviews was assessed using the AMSTAR 2 tool [19]. Two reviewers (P.T. and E.S.) independently performed the appraisal, and disagreements were resolved by consensus. The results were used to inform interpretation and sensitivity analyses. Sensitivity analyses were conducted by restricting the meta‑analysis to reviews rated “High” or “Moderate” on AMSTAR 2 (excluding “Low” and “Critically Low” reviews).

### 2.6 Statistical Analysis

All analyses were conducted using R version 4.5.2 within RStudio (version 2024.12.1). A combined descriptive and quantitative synthesis approach was applied. Descriptive statistics were used to summarize characteristics of the included reviews, and a narrative synthesis was conducted where quantitative pooling was not appropriate. To assess overlap of primary studies across reviews, a citation matrix was constructed, and the CCA was calculated [28], with interpretation using established thresholds (< 0.05 = very low overlap; 0.05–0.10 = low overlap; 0.10–0.15 = moderate overlap; > 0.15 = high overlap).

For exposure–outcome pairs with sufficient data and low overlap, pooled effect sizes were recalculated using random‑effects meta‑analysis of meta‑analyses. The criteria for “sufficient data and low overlap” were: (1) at least three SRMAs reporting the exposure–outcome pair, (2) CCA < 0.05, and (3) sufficient reporting of effect estimates and standard errors for pooling. Effect sizes were extracted on the log scale and analyzed using the restricted maximum likelihood (REML) estimator [29] implemented in the metafor package (version 4.6‑0) [30]. Results were back‑transformed and reported as odds ratios with 95% confidence intervals.

Heterogeneity was assessed using Cochran's Q test, the I^2^ statistic, and τ^2^ [31]. Prediction intervals were calculated to estimate the expected range of effects in future studies [32]. Sensitivity analyses were performed by restricting analyses to reviews of higher methodological quality (as described in Section 2.5). Meta‑regression analyses were conducted to explore potential sources of heterogeneity, including publication year, number of included studies, and methodological quality. Statistical significance was set at *p* < 0.05 (two‑sided). Forest plots were generated using the metafor package (version 4.6‑0) [30] and ggplot2 (version 3.5.0).

### 2.7 Evidence Classification

- The overall strength of evidence was graded using established umbrella review criteria [20, 32]. Associations were classified into five levels based on statistical significance, sample size, heterogeneity, and robustness indicators:
- Class I (Convincing): *p* < 10^−6^, > 1000 cases, I^2^ < 50%, 95% prediction interval excluding the null, no evidence of small‑study effects, and no excess of statistical significance.
- Class II (Highly Suggestive): *p* < 10^−6^, > 1000 cases, largest study with *p* < 0.05.
- Class III (Suggestive): *p* < 10^−3^, > 1000 cases.
- Class IV (Weak): *p* < 0.05 but does not meet higher class criteria.
- Class V (Non‑significant): *p* ≥ 0.05.

### 2.8 Publication Bias

Assessment of publication bias was not performed, as none of the pooled analyses included 10 or more studies, limiting the reliability of funnel plot‑based methods [33].

## 3 Results

### 3.1 Study Selection

We identified 1770 records from the database searches. After removing 457 duplicates, 1313 records were screened by title and abstract, of which 1291 were excluded. The remaining 23 full‑text articles and the two articles identified by citation searching were assessed for eligibility. Nine out of those 25 articles were excluded after full‑text screening (reasons in Figure 1), leaving 16 SRMAs that met the inclusion criteria. The flow diagram is presented in Figure 1.

**Figure 1:**
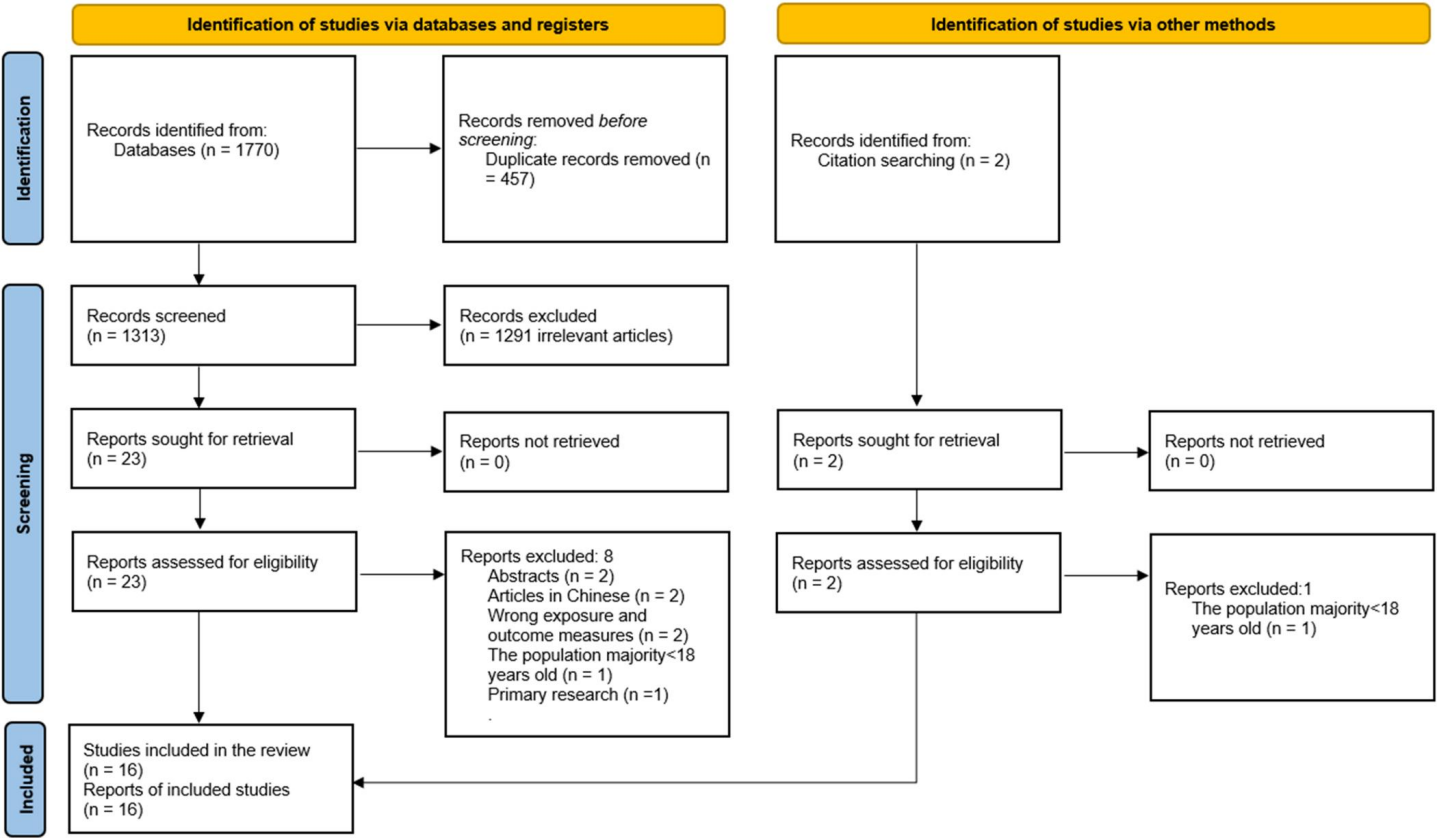
PRISMA 2020 flow diagram. PRISMA 2020 flow diagram of study selection for the umbrella review. Records were identified through database searching (*n* = 1770). After removing duplicates (*n* = 457), 1313 records were screened by title and abstract, of which 1291 were excluded. The remaining 23 full‑text articles and two articles identified by citation searching were assessed for eligibility. Nine out of those 25 articles were excluded after full‑text screening, leaving 16 systematic reviews with meta‐analyses (SRMAs) that met the inclusion criteria.

### 3.2 Characteristics of Included Reviews

The 16 SRMAs were published between 2008 and 2024, all focusing on adult populations (≥ 18 years). The total number of participants summed across reviews was approximately 13.8 million (range 275–5,172,718). The 16 SRMAs examined a range of sleep exposures. Short sleep duration was the most frequently examined exposure, followed by long sleep duration. Other exposures (daytime napping, social jetlag, insomnia, sleep restriction) were each examined in a single SRMA. Obesity outcomes included BMI‑defined overweight/obesity, central obesity (waist circumference, waist‑to‑hip ratio), weight gain, and body fat. A detailed summary of each SRMA is provided in Table 1.

**Table 1:** Characteristics of included systematic reviews and meta‐analyses.

First Author (Year) [Ref #]	N Studies	N Participants (Total)	Primary Study Designs	Sleep Exposures Covered	Obesity Outcomes Covered	Key Subgroup/Notes
Sperry (2015) [9]	21	56,259	Cross‑sectional, Cohort	Short sleep duration (< 5–6 h vs 7–8 h); continuous sleep duration	Central obesity (WC binary and continuous)	Meta‑regression: age trend (*p* = 0.05); sex not significant
Cai (2023) [34]	9	167,031	Cross‑sectional, cohort	Daytime napping (any vs none; duration < 1 h vs > 1 h)	Obesity (BMI ≥ 25, 28, 30)	Adult‑only meta‑analysis; subgroups: nap > 1 h (OR 1.32), BMI ≥ 30 (OR 1.43), geographic variation
Jiang (2024) [35]	15	52,318	Cross‑sectional (12), Cohort (3)	Short sleep (< 6–7 h), long sleep (> 8–9 h)	Obesity (BMI‑defined, varied cutoffs)	Subgroup: BMI ≥ 27 gave OR 2.41; objective sleep OR 2.70
Cappuccio (2008) [36]	45	634,511	Cross‑sectional, Cohort	Short sleep (< 5 h)	Obesity (BMI ≥ 30), BMI (continuous)	Also reported per‑hour sleep reduction (β = –0.35 kg/m^2^)
Itani (2017) [8]	108	5,172,718	Prospective cohort	Short sleep (< 5 h, < 6 h, < 7 h)	Obesity incidence (various BMI cutoffs)	Meta‑regression showed shorter sleep associated with decrease in obesity (coef=0.181, *p* = 0.042)
Kohanmoo (2024) [37]	7	195,555	Prospective cohort	Short sleep (≤ 5–7 h), long sleep (> 8–9 h)	Central obesity (WC, WHR)	Short sleep significant (RR 1.08); long sleep non‑significant (RR 1.02)
Jike (2018) [10]	95 (15 for obesity)	5,134,036	Prospective cohort	Long sleep (> 7–10 h)	Obesity (BMI ≥ 25 or ≥ 30, WC, weight gain)	Subgroup: age < 65 y RR 1.09; females RR 1.12 (borderline)
Guimarães (2022) [38]	36 (22 for obesity)	488,488	Cohort	Short sleep (< 7 h)	Obesity (overweight, obesity, abdominal, weight gain)	Dose‑response: per 1 h decrease OR 1.26; highest risk for < 5 h (OR 1.38)
Zhou (2019) [39]	12	198,579	Prospective cohort	Short sleep, long sleep	Obesity (BMI ≥ 30 or ≥ 25)	Per 1 h decrement RR 1.09; long sleep non‑significant
Wu (2014) [7]	11	197,906	Prospective cohort	Short sleep (≤ 5–6 h), long sleep (≥ 7–9 h)	Obesity (BMI ≥ 30 or ≥ 25)	Subgroup: men OR 1.65; ≤ 5 h OR 1.32; ≤ 6 h OR 2.74
Lange (2024) [40]	6	132,472	Longitudinal cohort	Short sleep (< 5 h)	Obesity incidence (BMI ≥ 24–30)	No sex difference (ROR 1.04); women OR 1.36, men OR 1.26
Yu (2019) [41]	6	275	RCT	Sleep restriction (≤ 5–6 h vs 6–9 h)	Body weight (kg), body fat	Weight gain non‑significant (SMD 0.44); body fat non‑significant (SMD 0.35)
Chan (2018) [14]	67	~654,108^a^	Cross‑sectional, Longitudinal	Insomnia diagnosis (IND), insomnia symptoms (INS)	Obesity, overweight, BMI (mean difference, correlation)	INS‑BMI correlation r = 0.06 (weak); IND not associated with obesity
Liu (2019) [42]	16	329,888	Prospective cohort	Long sleep (> 7–10 h)	Obesity (BMI ≥ 30/25), weight gain (≥ 5 kg), BMI change, weight change	Obesity RR 1.04; weight gain non‑significant overall; BMI change β –0.001
Arab (2024) [16]	43	231,648	Cross‑sectional, Longitudinal, Case‑control	Social jetlag (MSF–MSW)	BMI (continuous)	r = 0.11 (95% CI 0.03–0.19), high heterogeneity (I^2^ = 97.2%)
Bacaro (2020) [12]	12	154,936	Longitudinal, Prospective cohort	Short sleep (< 5–6.5 h), long sleep (> 7–9 h)	Obesity (BMI‑defined)	Short sleep OR 1.412; extreme short sleep (≤ 5 h) OR 1.500; long sleep non‑significant

Abbreviations: β, regression coefficient; BMI, body mass index; CI, confidence interval; IND, insomnia diagnosis; INS, insomnia symptoms; MSF, mid‑sleep on free days; MSW, mid‑sleep on work days; OR, odds ratio; RCT, randomized controlled trial; RR, relative risk; r, Pearson correlation coefficient; SMD, standardized mean difference; WC, waist circumference; WHR, waist‑to‑hip ratio.

^a^ Derived from the 13 IND studies (*n* = 27,986) and the 49 cross‑sectional INS studies (mean *n* = 12,778 × 49 ≈ 626,122). Longitudinal studies included in the review did not report participant totals.

### 3.3 Methodological Quality (AMSTAR 2)

Quality assessment using AMSTAR 2 [19] revealed that two reviews were rated High, four were Moderate, nine were Low, and one was Critically Low (Supplementary Table S2). The most common critical flaws were the absence of a protocol (item 2) and the lack of a list of excluded studies (item 7). Non‑critical weaknesses frequently included missing duplicate selection/extraction and incomplete reporting of funding sources.

### 3.4 Overlap of Primary Studies

The CCA was 0.024, indicating very low overlap of primary studies across the 16 SRMAs [28], supporting the independence of the evidence base. Pair‑specific analysis revealed a CCA of 0.063 for short sleep–obesity (low overlap) and 0.100 for long sleep–obesity (moderate overlap). The moderate overlap for long sleep suggests that the evidence base for this association is less independent than for short sleep, which should be considered when interpreting the results.

### 3.5 Narrative Synthesis of Individual SRMA Findings

For exposures with only a single SRMA available, findings are described narratively.

Daytime napping was associated with a 23% increased odds of obesity (OR 1.23, 95% CI 1.11–1.37) [34]. Subgroup analyses revealed that naps > 1 h were associated with a 32% increased risk (OR 1.32, 95% CI 1.19–1.47; *k* = 5), while naps < 1 h were not significant (OR 1.17, 95% CI 0.98–1.39; *k* = 4) [34]. The association was strongest when obesity was defined as BMI ≥ 30 (OR 1.43, 95% CI 1.24–1.65) and weaker when defined as BMI ≥ 25 (OR 0.85, 95% CI 0.73–1.00). Geographic variation was notable, with the highest odds in the UK (OR 1.39), followed by the USA (OR 1.27), and China (OR 1.05); a single Spanish study reported an extreme effect (OR 9.36, 95% CI 4.74–18.45) [34].

Social jetlag showed a small positive correlation with BMI (*r* = 0.11, 95% CI 0.03–0.19) [16].

Insomnia symptoms were weakly correlated with BMI (*r* = 0.06, 95% CI 0.01–0.12; *p* = 0.03), while insomnia diagnosis was not significantly associated with obesity [14].

Sleep restriction in randomized controlled trials did not lead to significant weight gain (SMD 0.44, 95% CI –0.13 to 1.02; *k* = 6) [41].

A comprehensive summary of subgroup findings from individual SRMAs, including age, sex, geographic region, sleep measurement, and dose‑response analyses, is provided in Supplementary Table S3.

### 3.6 Quantitative Synthesis of Pooled Findings

#### 3.6.1 Main Pooled Findings and Evidence Classification

Short sleep duration was associated with a significantly increased risk of obesity in the random‑effects meta‑analysis of seven SRMAs: pooled OR = 1.40 (95% CI 1.27–1.55; *p* < 0.001), with substantial heterogeneity (I^2^ = 83.7%) [31]. The prediction interval (1.02–1.92) suggests the effect is likely to be positive in future studies [32] (Figure 2, Table 2).

**Figure 2:**
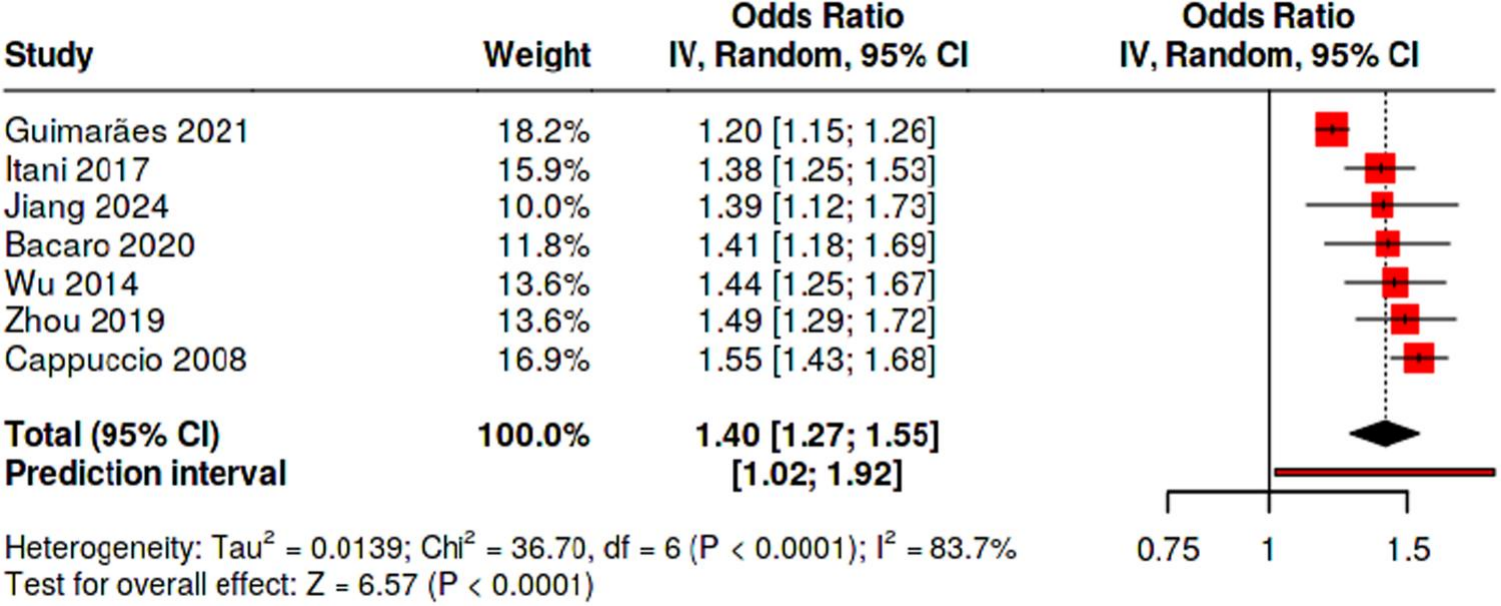
Forest plot – short sleep duration and obesity risk. Forest plot of the association between short sleep duration and obesity risk in adults. Each square represents the odds ratio (OR) from an individual systematic review with meta‑analysis. The size of each square is proportional to the weight assigned to that review in the random‑effects model. Horizontal lines represent the 95% confidence intervals (CIs). The diamond at the bottom represents the pooled OR with its 95% CI. The pooled OR was 1.40 (95% CI 1.27–1.55), with substantial heterogeneity (I^2^ = 83.7%). The prediction interval (1.02–1.92) suggests the effect is likely to be positive in future studies. CI = confidence interval, OR = odds ratio.

**Table 2:** Summary of pooled findings (main exposures).

Exposure	*k*	Effect estimate	95% CI	I^2^ (%)	*p* value	Prediction interval	Evidence class (Ioannidis [20])
Short sleep	7	1.40	1.27–1.55	83.7%	< 0.0001	1.02–1.92	Class III (Suggestive)
Long sleep	7	1.05	1.02–1.08	0.0%	0.0005	1.01–1.09	Class III (Suggestive)
Daytime napping	1	1.23	1.11–1.37	—	< 0.0001	—	Class IV (Weak)
Social jetlag	1	1.12 (r)	1.03–1.21	—	—	—	Class IV (Weak)
Insomnia symptoms	1	*r* = 0.06	0.01–0.12	—	0.03	—	Class IV (Weak)

*Note:* Pooled odds ratios (OR) for short and long sleep were calculated using random‑effects meta‑analysis (REML). For exposures with only one meta‑analysis, the original effect measure is reported (r for social jetlag and insomnia symptoms; OR for daytime napping). The prediction interval (PI) estimates the range in which the true effect of a future study would fall, accounting for heterogeneity [32]. Evidence classification follows Ioannidis criteria [20]: Class III (Suggestive) – *p* < 10^−3^ and > 1000 cases; Class IV (Weak) – *p* < 0.05 but does not meet higher class criteria. For *k* = 1 rows, these are single‐review estimates (not pooled).

Long sleep duration was also associated with a modestly increased risk in seven SRMAs: pooled OR = 1.05 (95% CI 1.02–1.08; *p* = 0.0005), with no heterogeneity (I^2^ = 0%). The prediction interval (1.01–1.09) indicates a consistent small effect (Table 2, Figure 3).

**Figure 3:**
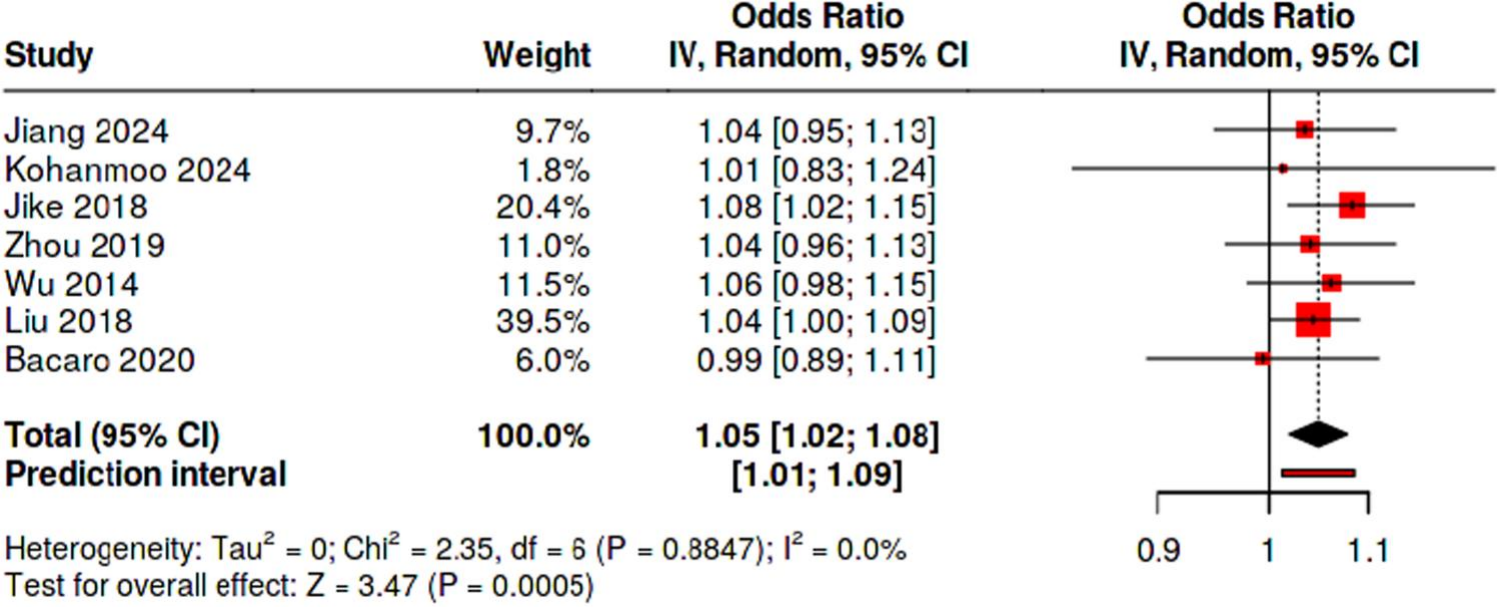
Forest plot – long sleep duration and obesity risk. Forest plot of the association between long sleep duration and obesity risk in adults. Each square represents the odds ratio (OR) from an individual systematic review with meta‑analysis. The size of each square is proportional to the weight assigned to that review in the fixed‑effects model (no significant heterogeneity was detected). Horizontal lines represent the 95% confidence intervals (CIs). The diamond at the bottom represents the pooled OR with its 95% CI. The pooled OR was 1.05 (95% CI 1.02–1.08), with no heterogeneity (I^2^ = 0%). The prediction interval (1.01–1.09) indicates a consistent small effect. CI = confidence interval, OR = odds ratio.

Using the Ioannidis criteria [20], the associations for short and long sleep were classified as Class III (Suggestive), while daytime napping, social jetlag, and insomnia symptoms met only Class IV (Weak) evidence (Table 2).

#### 3.6.2 Subgroup Analyzes

##### 3.6.2.1 Sleep Cutoff

Subgroup analysis by sleep cutoff for short sleep showed a dose‑response pattern: ≤ 5 h (OR 1.53, 95% CI 1.42–1.64; *k* = 2, I^2^ = 0%), ≤ 6 h (OR 1.45, 1.25–1.68; *k* = 1), and ≤ 7 h (OR 1.29, 1.14–1.46; *k* = 3, I^2^ = 75.3%) (Table 3).

**Table 3:** Subgroup analysis by sleep cutoff (short sleep).

Cutoff	*k*	Pooled OR	95% CI	I^2^ (%)
≤ 5 h	2	1.53	1.42–1.64	0
≤ 6 h	1	1.45	1.25–1.68	0
≤ 7 h	3	1.29	1.14–1.46	75.3

*Note:* Cutoff groups are: ≤ 5 h (studies defining short sleep as ≤ 5 h); ≤ 6 h (≤ 5–6 h); ≤ 7 h (< 7–6.5 h). Pooled ORs were obtained using random‑effects meta‑analysis (REML).

##### 3.6.2.2 Age

The association between short sleep and obesity was stronger in younger adults. Itani et al. [8] found a significant effect in participants < 65 years (RR 1.43, 95% CI 1.24–1.64) but not in those ≥ 65 years; Bacaro et al. [12] reported a similar pattern for participants < 50 years (OR 1.75, 95% CI 1.29–2.36) versus ≥ 50 years (OR 1.09, 95% CI 0.79–1.51). Sperry et al. [9] noted a borderline trend with mean age (*p* = 0.05) in meta‑regression.

##### 3.6.2.3 Sex

Sex differences were inconsistent. Wu et al. [7] reported a stronger effect in men (OR 1.65, 95% CI 1.24–2.19) than in women (OR 1.25, 95% CI 1.06–1.47). In contrast, Lange et al. [40] found no significant sex difference (ratio of odds ratios 1.04, 95% CI 0.79–1.36). Jike et al. [10] observed a borderline effect in females (RR 1.12, 95% CI 1.00–1.26) but not in males.

##### 3.6.2.4 Geographic Region

For short sleep, Wu et al. [7] reported consistent effects across North America (OR 1.46, 95% CI 1.11–1.94), Europe (OR 1.45, 95% CI 0.79–2.64), and Japan (OR 1.47, 95% CI 1.16–1.87). Zhou et al. [39] noted that heterogeneity in the association between short sleep and obesity was partly explained by Asian versus Western populations, though separate estimates were not provided.

##### 3.6.2.5 Sleep Measurement

In one review limited to older adults (≥ 60 years), objective sleep measurement (polysomnography) was associated with a substantially larger risk (OR 2.70, 95% CI 1.80–4.05) than self‑report (OR 1.14, 95% CI 0.98–1.33) [35].

##### 3.6.2.6 Dose‑Response

A dose‑response relationship was consistently reported. Guimarães et al. [38] found that each 1‑h decrease in sleep duration increased obesity risk by 26% (OR 1.26, 95% CI 1.17–1.36). Sperry et al. [9] reported a pooled correlation of *r* = –0.09 (95% CI –0.14 to –0.03) for the continuous association between sleep duration and waist circumference. Cappuccio et al. [36] estimated that a 1‑h reduction in sleep was associated with a 0.35 kg/m^2^ increase in BMI (β = –0.35, 95% CI –0.57 to –0.12), equivalent to approximately 1.4 kg weight gain for a person of average height.

#### 3.6.3 Sensitivity Analysis and Meta‑Regression

We pre‑specified a sensitivity analysis restricted to SRMAs rated “High” (low risk of bias) on AMSTAR 2. As only one SRMA met this criterion, we broadened the analysis to include SRMAs rated “High” or “Moderate” (excluding only high or critically low risk of bias). For short sleep, the pooled OR for the four SRMAs with low/moderate risk of bias was 1.34 (95% CI 1.18–1.53; I^2^ = 74.2%), consistent with the primary estimate (OR 1.40). For long sleep, the three SRMAs with low/moderate risk of bias gave a pooled OR of 1.04 (95% CI 0.98–1.10; I^2^ = 0%), closely mirroring the primary estimate (OR 1.05). These findings support the robustness of our primary results.

Meta‑regression showed that publication year was a significant negative moderator (β = –0.013, 95% CI –0.022 to –0.004, *p* = 0.005), indicating that older reviews tended to report larger odds ratios. Number of primary studies and quality group were not significant (*p* = 0.73 and *p* = 0.18, respectively).

## 4 Discussion

### 4.1 Main Findings

This umbrella review synthesized evidence from 16 SRMAs encompassing approximately 13.8 million participants to provide a comprehensive evaluation of the association between sleep characteristics and obesity in adults. Short sleep duration emerged as the most robust sleep‑related risk factor, with a pooled odds ratio of 1.40 (95% CI 1.27–1.55) and a prediction interval entirely above unity. Long sleep duration was also associated with obesity, though the effect was modest (OR 1.05, 95% CI 1.02–1.08). Both associations were classified as Class III (Suggestive) under the Ioannidis criteria [20]. In contrast, daytime napping [34], social jetlag [16], insomnia symptoms [14], and experimental sleep restriction [41] showed only weak or non‑significant associations, meeting Class IV (Weak) evidence at best.

### 4.2 Mechanistic Interpretation

The suggestive association between short sleep and obesity is supported by a substantial body of mechanistic evidence. Experimental studies demonstrate that sleep curtailment suppresses the anorexigenic hormone leptin while elevating orexigenic ghrelin, creating a hormonal milieu that promotes hunger and caloric intake [43, 44]. Although short sleep marginally increases basal energy expenditure, it disproportionately enhances the hedonic drive for energy‑dense foods, resulting in a net positive energy balance [45, 46]. These neuroendocrine and behavioral pathways are further reinforced by dysregulation of glucose metabolism and increased sympathetic nervous system activity [6, 47].

Recent evidence suggests a bidirectional relationship between obesity and sleep‑disordered breathing, with metabolic pathways linking adipose tissue dysfunction to upper airway collapse [48]. This reciprocal interaction underscores the complexity of the sleep‑obesity relationship and highlights the need for integrated preventive and therapeutic approaches.

Notably, our synthesis supports a J‑shaped rather than a symmetrical U‑shaped association: the obesity risk for short sleep substantially outweighed that for long sleep. This asymmetry suggests that while short sleep functions as a primary metabolic driver, long sleep may largely represent an epiphenomenon or surrogate marker for residual confounding by chronic illness, depression, or low socioeconomic status [49, 50]. The non‑significant findings from the experimental meta‑analysis by Yu et al. [41] reinforce this interpretation; the limited duration of randomized trials (typically days to weeks) is unlikely to capture the cumulative metabolic effects of chronic sleep restriction observed in longitudinal cohorts.

### 4.3 Directional Consistency and Emerging Sleep Constructs

A notable finding of this umbrella review is the remarkable directional consistency of the short sleep–obesity association across the included SRMAs. Despite substantial variation in geographic setting, demographic composition, outcome definitions, and analytical approaches, the vast majority of reviews reported positive associations of similar magnitude. This degree of reproducibility across methodologically heterogeneous reviews is a hallmark of a robust epidemiological relationship and supports its Class III classification. By contrast, the evidence for long sleep was characterized by marginal effect sizes and limited biological plausibility as a direct causal pathway, consistent with recent large‑scale cohort studies that question the symmetry of the traditional U‑curve [42, 50].

For emerging sleep constructs—daytime napping [34], social jetlag [16], and insomnia [14]—each was represented by only a single meta‑analysis in our review, precluding a definitive meta‑synthesis and underscoring these as priority areas for future systematic review activity. The identification of these evidence gaps is itself a valuable contribution, as it directs researchers toward constructs where the current evidence base remains insufficient to draw firm conclusions.

### 4.4 Heterogeneity and Methodological Considerations

The substantial heterogeneity observed for short sleep (I^2^ = 83.7%) reflects several methodological sources of variance in the underlying literature rather than true inconsistency in the direction of association. The most important source is the lack of standardized exposure thresholds, which ranged from ≤ 5 to < 7 h across reviews. Our subgroup analysis demonstrated a clear dose–response gradient: ≤ 5 h (OR 1.53, 95% CI 1.42–1.64; I^2^ = 0%), ≤ 6 h (OR 1.45, 95% CI 1.25–1.68; I^2^ = 0%), and ≤ 7 h (OR 1.29, 95% CI 1.14–1.46; I^2^ = 75.3%). This gradient both explains a portion of this heterogeneity and strengthens the case for a consistent association under the Bradford Hill criteria [51].

The mode of sleep assessment was an equally critical moderator: objective measurements (polysomnography) yielded an OR of 2.70 (95% CI 1.80–4.05) compared with 1.14 (95% CI 0.98–1.33) for self‑report [35]. However, this finding is based on a single meta‑analysis and requires further investigation before definitive conclusions can be drawn. Nonetheless, it suggests that recall bias in survey‑based studies may systematically attenuate the true association and that the real magnitude of risk could be larger than current pooled estimates indicate.

### 4.5 Quality of Evidence and Meta‑Regression

The very low overall CCA of 0.024 confirmed that the included SRMAs drew on largely independent primary evidence, minimizing the risk of double‑counting that can inflate confidence in umbrella reviews [28]. However, the pair‑specific CCA for long sleep–obesity (0.100) suggested moderate overlap, indicating that the evidence base for this association is less independent than for short sleep–obesity (0.063) and should be interpreted with greater caution.

Methodological quality, assessed using AMSTAR 2 [19], remains a concern: the majority of included SRMAs were rated Low or Critically Low, primarily due to the absence of registered protocols and lists of excluded studies. Importantly, the sensitivity analysis restricted to higher‑quality reviews (*k* = 4) yielded a pooled OR of 1.34 (95% CI 1.18–1.53), consistent with the primary estimate and with substantial heterogeneity (I^2^ = 74.2%), suggesting that the overall finding for short sleep is not driven by lower‑quality reviews.

Meta‑regression identified publication year as a significant negative moderator (β = –0.013, *p* = 0.005), indicating that older reviews tended to report larger effect sizes. This may reflect improvements in confounder adjustment over time, more conservative exposure definitions in recent primary studies, or changes in study design and reporting standards. The observed trend highlights the importance of considering the evolving methodological quality of the evidence base when interpreting pooled estimates, as findings from older reviews may overestimate the true association.

### 4.6 Comparison with Other Umbrella Reviews

Only two prior umbrella reviews have examined sleep and obesity at the synthesis‑of‑syntheses level. Gao et al. reported that short sleep was associated with increased obesity risk but did not provide a pooled estimate specifically for obesity, included mixed child/adult populations, and did not quantify primary study overlap or grade evidence [18].

Benfica et al. conducted a comprehensive overview of meta‐analyses examining the relationship between sleep duration and overweight/obesity [17]. They reported that short sleep duration increased the risk of overweight/obesity in adults (RR 1.30; 95% CI 1.17–1.44), which is consistent with our pooled estimate (OR 1.40). For long sleep duration, they found a modest increased risk in adults (RR 1.05; 95% CI 1.02–1.08), also consistent with our findings (OR 1.05). However, their overview included mixed populations (children, adolescents, and adults), used the ROBIS tool for quality assessment rather than AMSTAR 2, and did not grade evidence using Ioannidis criteria or calculate CCA.

Our review extends these prior syntheses by: (i) focusing exclusively on adults; (ii) covering the full spectrum of sleep exposures (duration, quality, disorders, chronotype); (iii) quantifying primary study overlap (overall CCA = 0.024; short sleep–obesity CCA = 0.063; long sleep–obesity CCA = 0.100); (iv) applying AMSTAR 2 quality appraisal; and (v) grading evidence using Ioannidis criteria [20]. Like prior reviews, we confirm short sleep as the most robust obesity risk factor. However, our review adds that short sleep is only Class III (Suggestive), long sleep is Class III (likely residual confounding), and other sleep exposures (napping, social jetlag, insomnia) have only Class IV (Weak) evidence, highlighting critical research gaps. The moderate overlap observed for long sleep (CCA = 0.100) suggests that the evidence base for this association is less independent than for short sleep, which should be considered when interpreting findings.

### 4.7 Strengths

This umbrella review has several methodological strengths. The umbrella design provides the highest level of observational evidence synthesis by integrating results across multiple independent meta‐analyses. The large cumulative sample size enhances statistical power and generalizability. The very low overall CCA (0.024) confirms that the included reviews drew on largely independent primary evidence, minimizing the risk of double‑counting that can inflate confidence in umbrella reviews [28]. The use of random‑effects meta‑analysis of meta‐analyses with prediction intervals provides both a summary estimate and a realistic range for future study effects [32]. The application of the AMSTAR 2 tool [19] for quality appraisal and the Ioannidis framework [20] for evidence grading ensures a transparent and reproducible assessment of both individual review quality and overall evidence credibility. Furthermore, this is the first umbrella review to focus exclusively on adult populations while covering the full spectrum of sleep exposures and incorporating formal overlap quantification.

### 4.8 Limitations

Several limitations warrant consideration. First, the evidence is derived predominantly from observational studies, precluding definitive causal inference despite the consistency and dose–response pattern of the associations. Second, the substantial heterogeneity for short sleep, while partly explained by definitional and methodological variation, indicates residual unexplained variance that may reflect population‑level differences in confounding structures. Third, the widespread reliance on self‑reported sleep measures in the underlying primary studies likely introduces misclassification bias that attenuates effect estimates, as demonstrated by the markedly larger effects observed with objective measurement in a single review [35]. Fourth, for several sleep exposures—daytime napping, social jetlag, insomnia, and sleep restriction—only a single meta‑analysis was available, limiting the breadth and robustness of evidence synthesis for these domains. Fifth, formal assessment of publication bias was not performed because none of the pooled analyses included ten or more studies, the minimum threshold for reliable funnel plot–based methods [33]. Sixth, while subgroup data on age, sex, and geographic region were available from individual reviews, these were reported inconsistently and could not be formally pooled at the umbrella level. Finally, the moderate overlap observed for long sleep–obesity (CCA = 0.100) suggests some redundancy in the primary evidence informing this association, and findings for this outcome should be interpreted with greater caution.

### 4.9 Clinical Implications

The findings carry important clinical and public health implications. The consistent, suggestive‑level association between short sleep and obesity positions sleep duration as a potentially modifiable risk factor that warrants consideration in obesity prevention and management frameworks. Incorporating sleep assessment into routine clinical encounters, particularly in primary care and metabolic health settings, could improve risk stratification and support more comprehensive lifestyle counseling [6, 52].

At the population level, given that more than one‑third of adults sleep less than the recommended duration [4], even modest improvements in population sleep habits could yield meaningful reductions in obesity prevalence. The dose–response gradient observed in our analysis suggests that clinical messaging need not focus solely on achieving optimal sleep duration; incremental improvements in sleep time may confer proportional reductions in obesity risk.

However, as the evidence is observational and classified only as Class III (Suggestive), these implications should be considered preliminary, and clinicians should balance this information with other established risk factors for obesity.

### 4.10 Future Research Directions

Future research should address four critical gaps. First, the adoption of standardized sleep duration thresholds across studies would reduce heterogeneity and improve comparability. Second, greater use of objective sleep measurement (actigraphy, polysomnography) would clarify the true magnitude of the sleep–obesity association and reduce misclassification bias. Third, long‑term longitudinal studies and pragmatic randomized trials of sleep extension interventions are needed to establish whether improving sleep duration causally reduces obesity incidence and to quantify the magnitude of benefit achievable. Given the observational nature of the evidence, randomized controlled trials of sleep extension would provide the strongest test of causality, though such trials face practical challenges in implementation and long‑term follow‑up. Fourth, the emerging sleep constructs identified as having only weak evidence in this review—social jetlag, insomnia, and daytime napping—require additional high‑quality systematic reviews before their role in obesity can be reliably determined.

## 5 Conclusion

This umbrella review provides the most comprehensive synthesis to date of the association between sleep characteristics and obesity exclusively in adults. Short sleep duration is the most consistently and strongly associated sleep‑related risk factor for obesity, supported by suggestive‑level evidence, a clear dose–response gradient, very low overlap across independent meta‐analyses, and robustness to sensitivity analysis by review quality. Long sleep duration shows a statistically significant but clinically marginal association that likely reflects residual confounding rather than a direct causal pathway. For other sleep exposures—daytime napping, social jetlag, insomnia, and experimental sleep restriction—the current evidence remains weak and insufficient to draw firm conclusions. The striking discrepancy between objective and self‑reported sleep measurement effects suggests that the true magnitude of the short sleep–obesity association may be substantially underestimated in the existing literature. These findings support the integration of sleep duration assessment into clinical obesity risk evaluation and population‑level prevention strategies, though causality cannot be established from the available evidence. To advance the evidence from suggestive to convincing, the field requires standardized exposure definitions, routine use of device‑based sleep measurement, pre‑registered systematic reviews, and pragmatic trials testing whether sleep extension interventions reduce obesity incidence in adults.

## Author Contributions

**Iana Malasevskaia:** conceptualization, investigation, writing – original draft, methodology, validation, visualization, writing – review and editing, software, formal analysis, project administration, data curation, supervision. **Delvy Rebellow:** data curation, investigation, formal analysis, writing – original draft. **Pakeeza Tarar:** investigation, writing – original draft, formal analysis, data curation. **Esraa M. Soliman:** conceptualization, validation, methodology, writing – review and editing.

## Funding

The authors have nothing to report.

## Disclosure

All authors have read and approved the final version of the manuscript. Iana Malasevskaia had full access to all of the data in this study and takes complete responsibility for the integrity of the data and the accuracy of the data analysis.

## Ethics Statement

Not applicable. This umbrella review synthesized data from published systematic reviews and meta‐analyses; no primary data were collected, and no human participants were involved.

## Consent

The authors have nothing to report.

## Conflicts of Interest

The authors declare no conflicts of interest.

## Declaration of Generative AI and AI‑Assisted Technologies in the Writing Process

During the preparation of this work, the author(s) used ChatGPT to improve language and readability. After using this tool, the author(s) reviewed and edited the content as needed and take(s) full responsibility for the content of the publication.

## Transparency Statement

Iana Malasevskaia affirms that this manuscript is an honest, accurate, and transparent account of the study being reported; that no important aspects of the study have been omitted; and that any discrepancies from the study as planned have been explained.

## Supporting information


Supporting File

## Data Availability

All data generated or analyzed during this study are included in this published article and its supplementary information files. No primary data were collected.
