# Supplementary material for: The Association Between Sleep and Obesity in Adults: An Umbrella Review of Meta‐Analyses
Source: Health Sci Rep. 2026 Oct 27;9(11):e73449. doi: 10.1002/hsr2.73449 (PMC13711346; doi:10.1002/hsr2.73449)
Supplement: Supplementary file 1 [file HSR2-9-e73449-s001.docx]

## **SUPPLEMENTARY MATERIAL**

### **Table S1. Database Search Records**

| Database | Search Strategy / Terms Used | Filters / Limits Applied | Date of Search | Results (n) |
| --- | --- | --- | --- | --- |
| PubMed/MEDLINE | (("Adult"[Mesh] OR "Adults"[Title/Abstract] OR "Adult"[Title/Abstract]) AND ("Sleep"[Mesh] OR "Sleep Duration"[Title/Abstract] OR "Sleep Quality"[Title/Abstract] OR "Short Sleep"[Title/Abstract] OR "Long Sleep"[Title/Abstract] OR "Sleep Disorders"[Mesh] OR "Insomnia"[Mesh] OR "Sleep Apnea, Obstructive"[Mesh] OR "Chronotype"[Title/Abstract]) AND ("Obesity"[Mesh] OR "Overweight"[Mesh] OR "Body Mass Index"[Mesh] OR "Waist Circumference"[Mesh] OR "Adiposity"[Mesh] OR "Obesity"[Title/Abstract] OR "Overweight"[Title/Abstract] OR "BMI"[Title/Abstract] OR "Waist Circumference"[Title/Abstract] OR "Central Obesity"[Title/Abstract] OR "Abdominal Obesity"[Title/Abstract])) | Full text, Meta-Analysis, Systematic Review, English | 23 Feb 2026 | 163 |
| Embase | ('adult'/exp OR 'adult':ab,ti OR 'adults':ab,ti) AND ('sleep'/exp OR 'sleep duration':ab,ti OR 'sleep quality':ab,ti OR 'short sleep':ab,ti OR 'sleep disorder'/exp OR 'insomnia'/exp OR 'obstructive sleep apnea'/exp OR 'chronotype':ab,ti) AND ('obesity'/exp OR 'overweight'/exp OR 'body mass'/exp OR 'waist circumference'/exp OR 'obesity':ab,ti OR 'overweight':ab,ti OR 'bmi':ab,ti OR 'waist circumference':ab,ti) AND ('meta analysis':ab,ti OR 'meta-analysis':ab,ti OR 'metaanalysis':ab,ti) | 'meta-analysis':ab,ti; English | 26 Feb 2026 | 781 |
| Cochrane CDSR | #1 ("adult" OR "adults") [956610]; #2 ("sleep" OR "sleep duration" OR "sleep quality" OR "sleep disorder" OR "insomnia" OR "sleep apnea") [73448]; #3 MeSH descriptor: [Sleep Wake Disorders] explode all trees [13237]; #4 MeSH descriptor: [Sleep Initiation and Maintenance Disorders] explode all trees [4222]; #5 MeSH descriptor: [Sleep] explode all trees [9423]; #6 MeSH descriptor: [Sleep Apnea Syndromes] explode all trees [4183]; #7 #2 OR #3 OR #4 OR #5 OR #6 [73944]; #8 ("obesity" OR "overweight" OR "BMI" OR "body mass index" OR "waist circumference" OR "adiposity") [142194]; #9 MeSH descriptor: [Obesity] explode all trees [22903]; #10 MeSH descriptor: [Overweight] explode all trees [26703]; #11 #8 OR #9 OR #10 [142291]; #12 #1 AND #7 AND #11 [4236] | Cochrane Reviews, English | 26 Feb 2026 | 304 |
| Scopus | TITLE-ABS-KEY(adult OR adults) AND TITLE-ABS-KEY(sleep OR "sleep duration" OR "sleep quality" OR insomnia OR "sleep apnea") AND TITLE-ABS-KEY(obesity OR overweight OR bmi OR "waist circumference") AND TITLE("meta analysis" OR "meta-analysis" OR metaanalysis) AND ( LIMIT-TO ( DOCTYPE , "re" ) ) AND ( LIMIT-TO ( LANGUAGE , "English" ) ) | LIMIT-TO (DOCTYPE, "re") [re = review]; English | 26 Feb 2026 | 178 |
| CINAHL (EBSCO) | (MH "Adult+") OR TI (adult OR adults) OR AB (adult OR adults) AND (MH "Sleep+") OR (MH "Sleep Disorders+") OR TI (sleep OR "sleep duration" OR "sleep quality" OR insomnia OR "sleep apnea" OR chronotype) OR AB (sleep OR "sleep duration" OR "sleep quality" OR insomnia OR "sleep apnea" OR chronotype) AND (MH "Obesity+") OR (MH "Body Mass Index+") OR (MH "Waist Circumference+") OR TI (obesity OR overweight OR BMI OR "waist circumference" OR adiposity) OR AB (obesity OR overweight OR BMI OR "waist circumference" OR adiposity) AND ("meta-analysis") | Meta Analysis | 1 Mar 2026 | 85 |
| Epistemonikos | (title:(adult OR adults) OR abstract:(adult OR adults)) AND (title:(sleep OR "sleep duration" OR "sleep quality" OR insomnia OR "sleep apnea") OR abstract:(sleep OR "sleep duration" OR "sleep quality" OR insomnia OR "sleep apnea")) AND (title:(obesity OR overweight OR BMI OR "body mass index" OR "waist circumference") OR abstract:(obesity OR overweight OR BMI OR "body mass index" OR "waist circumference")) | Systematic reviews | 23 Feb 2026 | 259 |
| TOTAL |  |  |  | 1,770 |

### **Table S2. AMSTAR 2 Quality Assessment of Included Reviews**

### **Part A. Item‑Level Assessment (Y = Yes, P = Partial Yes, N = No)**

| Author (Year) | Ref | 1 | 2 | 4 | 9 | 11 | 13 | 15 | 3 | 5 | 6 | 7 | 8 | 10 | 12 | 14 | 16 |
| --- | --- | --- | --- | --- | --- | --- | --- | --- | --- | --- | --- | --- | --- | --- | --- | --- | --- |
| Sperry (2015) | [11] | Y | N | N | Y | N | N | Y | P | Y | N | Y | Y | Y | Y | N | Y |
| Cai (2023) | [36] | Y | N | Y | Y | N | N | N | P | Y | Y | Y | Y | Y | Y | Y | Y |
| Jiang (2024) | [37] | Y | Y | Y | Y | Y | Y | Y | Y | Y | Y | N | Y | Y | Y | Y | Y |
| Cappuccio (2008) | [7] | Y | Y | Y | N | Y | Y | Y | Y | Y | Y | N | Y | N | Y | Y | Y |
| Itani (2017) | [10] | Y | N | Y | Y | N | Y | Y | Y | Y | Y | Y | Y | Y | Y | Y | Y |
| Kohanmoo (2024) | [38] | Y | Y | Y | Y | Y | N | Y | P | Y | Y | N | Y | Y | Y | Y | Y |
| Jike (2018) | [12] | Y | N | Y | Y | Y | N | Y | Y | Y | Y | Y | Y | Y | Y | Y | Y |
| Guimarães (2022) | [39] | Y | Y | Y | N | Y | N | Y | Y | Y | Y | Y | Y | Y | Y | Y | Y |
| Zhou (2019) | [40] | Y | N | Y | N | Y | Y | Y | P | Y | Y | Y | Y | N | Y | Y | Y |
| Wu (2014) | [9] | Y | N | N | Y | Y | Y | Y | Y | Y | Y | Y | Y | Y | Y | Y | Y |
| Lange (2024) | [41] | Y | Y | Y | Y | Y | N | Y | Y | Y | Y | N | Y | Y | Y | Y | N |
| Yu (2019) | [42] | Y | Y | Y | Y | N | N | Y | P | Y | Y | Y | Y | Y | Y | Y | Y |
| Chan (2018) | [16] | Y | N | Y | Y | N | N | Y | P | Y | Y | Y | Y | Y | Y | Y | Y |
| Liu (2019) | [43] | Y | N | Y | Y | N | Y | Y | Y | Y | Y | Y | Y | Y | Y | Y | Y |
| Arab (2024) | [18] | Y | Y | Y | Y | N | N | Y | Y | Y | Y | Y | Y | Y | Y | Y | Y |
| Bacaro (2020) | [14] | Y | Y | Y | Y | N | N | Y | Y | Y | Y | Y | Y | N | Y | Y | Y |

### Legend for Part A: Y = Yes; P = Partial Yes; N = No Critical domains (2, 4, 7, 9, 11, 13, 15): protocol, search, excluded list, RoB, meta‑analysis methods, RoB in discussion, publication bias Non‑critical domains (1, 3, 5, 6, 8, 10, 12, 14, 16): PICO, design justification, duplicate selection, duplicate extraction, study details, funding, RoB impact, heterogeneity explanation, COI

### **Part B. Overall Quality Rating**

| Author (Year) | Ref | Critical Flaws | Non‑Critical Weaknesses | Overall Rating |
| --- | --- | --- | --- | --- |
| Sperry (2015) | [11] | 3 | 3 | Critically Low |
| Cai (2023) | [36] | 1 | 3 | Low |
| Jiang (2024) | [37] | 0 | 1 | High |
| Cappuccio (2008) | [7] | 1 | 3 | Low |
| Itani (2017) | [10] | 1 | 0 | Low |
| Kohanmoo (2024) | [38] | 1 | 1 | Low |
| Jike (2018) | [12] | 1 | 1 | Low |
| Guimarães (2022) | [39] | 0 | 2 | Moderate |
| Zhou (2019) | [40] | 1 | 2 | Low |
| Wu (2014) | [9] | 2 | 0 | Moderate |
| Lange (2024) | [41] | 1 | 2 | Low |
| Yu (2019) | [42] | 0 | 1 | High |
| Chan (2018) | [16] | 1 | 2 | Low |
| Liu (2019) | [43] | 1 | 1 | Low |
| Arab (2024) | [18] | 0 | 2 | Moderate |
| Bacaro (2020) | [14] | 0 | 3 | Moderate |

### Summary of Overall Ratings:

| Rating | Number of Reviews |
| --- | --- |
| High | 2 |
| Moderate | 4 |
| Low | 9 |
| Critically Low | 1 |
| Total | 16 |

### Legend for Part B: High = 0–1 non‑critical weakness, no critical flaws Moderate = ≥2 non‑critical weaknesses, no critical flaws Low = ≥1 critical flaw Critically Low = ≥2 critical flaws

### **Table S3. Key Subgroup Findings from Individual Systematic Reviews with Meta‑Analyses**

| First Author (Year) | Ref | Subgroup Category | Subgroup Level | Effect Size (95% CI) | Interaction / Notes |
| --- | --- | --- | --- | --- | --- |
| Itani (2017) | [10] | Age | <65 years | RR 1.43 (1.24–1.64) | Significant; effect absent in ≥65y |
| Jiang (2024) | [37] | Sleep measurement | Objective (polysomnography) | OR 2.70 (1.80–4.05) | Much larger than self‑report (OR 1.14) |
| Wu (2014) | [9] | Sex | Men | OR 1.65 (1.24–2.19) | Stronger than women (OR 1.25) |
| Cai (2023) | [36] | Geographic region | UK | OR 1.39 (1.32–1.47) | Largest effect; China OR 1.05 |
| Lange (2024) | [41] | Sex | Men | OR 1.26 (1.13–1.40) | No significant sex difference (ROR 1.04) |
| Guimarães (2022) | [39] | Dose‑response | Per 1‑h decrease | OR 1.26 (1.17–1.36) | Consistent across 11 studies |
| Bacaro (2020) | [14] | Age | <50 years | OR 1.75 (1.29–2.36) | Significant; not in ≥50y |
| Zhou (2019) | [40] | Sex | Men | RR 1.12 (0.99–1.27) | Borderline; women RR 1.03 (0.92–1.15) |
| Cappuccio (2008) | [7] | Continuous sleep | Per 1‑h decrease in sleep | β = –0.35 kg/m² | Equivalent to ~1.4 kg weight gain for 178 cm person |

### Abbreviations: OR = odds ratio; RR = relative risk; β = regression coefficient (change in outcome per unit change in exposure); ROR = ratio of odds ratios (women/men or men/women); CI = confidence interval. Notes: Effect sizes are as reported in the original systematic reviews with meta‑analyses. "Per 1‑h decrease" refers to a one‑hour reduction in sleep duration per night. The β for Cappuccio (2008) [7] represents the change in BMI (kg/m²) per hour reduction; the approximate weight gain is calculated for a person of average height (178 cm). Subgroup findings are descriptive and not pooled across reviews.
